# Antioxidant Activities and Volatile Flavor Components of Selected Single-Origin and Blend Chocolates

**DOI:** 10.3390/molecules25163648

**Published:** 2020-08-11

**Authors:** Lucia Godočiková, Eva Ivanišová, Grzegorz Zaguła, Luis Noguera-Artiaga, Ángel A. Carbonell-Barrachina, Przemysław Łukasz Kowalczewski, Miroslava Kačániová

**Affiliations:** 1 Department of Microbiology, Faculty of Biotechnology and Food Sciences, Slovak University of Agriculture in Nitra, Tr. A. Hlinku 2, 94976 Nitra, Slovakia; 2 Department of Technology and Quality of Plant Products, Faculty of Biotechnology and Food Sciences, Slovak University of Agriculture in Nitra, Tr. A. Hlinku 2, 94976 Nitra, Slovakia; eva.ivanisova@uniag.sk; 3 Department of Bioenergy, Food Technology and Microbiology, Institute of Food Technology and Nutrition, University of Rzeszow, Cwiklinskiej 1, 35601 Rzeszow, Poland; g_zagula@ur.edu.pl (G.Z.); miroslava.kacaniova@gmail.com (M.K.); 4 Research Group “Food Quality and Safety”, Department of Agro-Food Technology, Miguel Hernández University of Elche, Carretera de Beniel, km 3.2, 03312 Orihuela, Alicante, Spain; lnoguera@umh.es (L.N.-A.); angel.carbonell@umh.es (Á.A.C.-B.); 5 Institute of Food Technology of Plant Origin, Poznań University of Life Sciences, 31 Wojska Polskiego St., 60-624 Poznań, Poland; przemyslaw.kowalczewski@up.poznan.pl; 6 Department of Fruit Sciences, Viticulture and Enology, Faculty of Horticulture and Landscape Engineering, Slovak University of Agriculture, Tr. A. Hlinku 2, 94976 Nitra, Slovakia

**Keywords:** antioxidant activity, aroma, blend, chocolate, ICP, flavonoids, polyphenols, single origin

## Abstract

The biological activity of chocolates gains more and more attention of consumers. Its antioxidant properties depend, among other factors, mainly on the origin of cocoa and the characteristics that this origin gives to the final product. Therefore, the aim of the study was to measure and compare the total content of polyphenols, antioxidant activity, and key odorants of commercial chocolates made from blend cocoa with single-origin ones. The highest content of polyphenols was found in 90% blend cocoa chocolate and single-origin samples, while the lowest content was exhibited by 100% chocolate from blend cocoa mass. The highest antioxidant activity measured by 2,2′-Azino-bis(3-ethylbenzothiazoline-6-sulfonic acid) diammonium salt (ABTS) and ferric reducing antioxidant power (FRAP) assays was observed in the sample of chocolate with 90% cocoa solids from blend mass, followed by single-origin chocolates. A high positive correlation between ABTS assay and the total polyphenol and phenolic acids’ content, as well as among the total content of polyphenols, flavonoids, and phenolic acids was found. Mineral composition analysis showed that dark chocolate is a valuable source of some elements, especially Mg, Fe, and Zn. Potentially toxic elements were not detected or below permitted limits. Moreover, it was noticed that the main volatile compound in all tested samples was acetic acid, but pyrazines were considered the most important group of chocolate odorants.

## 1. Introduction

Chocolate is a source of non-nutrient bioactive polyphenols with possible health benefits, including prebiotic activity and decreased risk of cardiovascular disease. The high phenolic capacity of cocoa contributes to the in vitro antioxidant activity compared to tea or red wine, which are generally described as polyphenol-rich products. However, comparing different studies and potential health benefits is difficult for the reason that not all polyphenols have the same bioactivity in vivo [1].

Chocolate is commonly prepared using a mix of cacao beans of different origins. However, single-origin chocolates are becoming continuously more popular and are frequently marketed as luxurious products, which arouses interest in the impact of cacao origin on the quality and food safety of the final product [2]. Single-origin chocolates are made from cocoa beans from selected crops, from a single plantation or farmer, or a given region. They are characterized by unique flavor and aroma derived from the cocoa beans used that are mainly determined by the geographical location of the plantation [3]. 

Various methods have been intensively studied to determine the antioxidant activities of samples. These methods differ in terms of their analysis principle and experimental conditions. Most of them are based on the investigation of a reaction in which a free radical is formed and how this reaction is inhibited by the addition of the sample extract [4]. The antioxidant capacity of foods can be evaluated by several available methods, the most prevalent being 2,2′-azino-bis(3-ethylbenzothiazoline-6-sulphonic acid) (ABTS) and 2,2-diphenyl-1-picrylhydrazyl (DPPH) assays. ABTS method was described as more sensitive to phenolic compounds and generally gives a lower analytical error than DPPH [5]. The antioxidant activity of cocoa beans varies depending on their origin [6]. 

Although chocolate is rich in polyphenols and other bioactive compounds, it also contains several minerals, specifically magnesium, potassium, copper, and iron [7]—in a wide range of concentrations. The content of minerals in chocolate depends on the amount of cocoa solids in the chocolate. Dark chocolate usually contains more minerals than milk chocolate due to the higher cocoa content in the composition [8]. Cocoa is a good source of magnesium, which is essential in catalyzing many biological reactions. It takes part in the synthesis of proteins, conduction of nerve impulses, muscle relaxation, and energy production [9]. Potassium, on the other hand, is essential for maintaining cell osmolarity and cell membrane potentials, but also plays a role in various biochemical pathways related to the proper functioning of the circulatory system and in controlling blood glucose levels as well [10]. The copper present in chocolate is involved in many enzymatic reactions, including the synthesis of collagen and neurotransmitters, as well as other trace minerals. Copper deficiencies in early development have been shown to cause cardiovascular disorders, but it is also indicated that too little dietary copper intake may contribute to vascular disease later in life [11]. Because metals can have both positive and negative roles in the human body, it is important to identify them in foodstuffs to understand their impact on human health [12].

Cocoa bean and chocolate quality are made up not only from mentioned attributes, but also the flavor volatiles are one of the most important components affecting cocoa bean and chocolate acceptability [13]. Raw cocoa beans exhibit an unpleasant tartness and bitterness. The desired cocoa flavor profile develops in the post-harvest stages of processing the beans, which mainly include fermentation, drying, and roasting [14]. Due to the many factors affecting the cocoa beans during processing, it is possible to impart a variety of flavor and quality to the cocoa obtained [15,16]. From a technological point of view, conching is the most important step in the production of chocolate, affecting not only its texture, but also its aroma [17]. This process improves the flavor profile and reduces the concentration of free acids and other volatile cocoa by-products [18]. Many studies have investigated the flavor composition of cocoa and chocolate products. The cocoa flavor of chocolate is influenced by both non-volatile and volatile chemical components. According to Afoakwa et al. [14], the key odor-active compounds in chocolate are primarily pyrazines, aldehydes, esters, alcohols, acids, and hydrocarbons. The 2-methylpropanal, 3-methylbutanal, phenylacetaldehyde, 2-ethyl-3,5-dimethylpyrazine, tetramethylpyrazine, trimethylpyrazine, 2-acetyl-1-pyrroline, 3-methylbutanoic acid, 3,5-diethyl-2-methylpyrazine,2-methylbutanal, and furaneol are considered the main factors influencing the overall flavor profile of cocoa products, with the first eight having the greatest impact on the flavor of chocolate [14,15]. Besides cocoa, also other ingredients of chocolate can influence its aroma, both by providing flavor compounds as well as precursor for other reactions, e.g., Maillard [19,20,21].

Therefore, the aim of the present study was to assess and compare the content of total polyphenols, flavonoids, and phenolic acids, as well as antioxidant activity, chemical elements, and aroma composition of blend cocoa chocolates and single-origin chocolates. 

## 2. Results and Discussion

### 2.1. Determination of Total Polyphenols, Flavonoids, and Phenolic Acids in Chocolates

Cocoa beans, and consequently also chocolate, are rich sources of antioxidant compounds, mainly catechins or flavan-3-ols, anthocyanins, and proanthocyanidins [22]. The primary catechin is (‒)-epicatechin and according to Nazaruddin et al. [23] its contents in cacao beans and powder were 4.61 and 3.81 mg/g, respectively, while total polyphenols were 34–60 and 20–62 mg/g (respectively). The anthocyanin fraction consists mainly of cyanidin-3-α-l-arabinoside and cyanidin-3-β-d-galactoside, while procyanidins are mostly flavan-3,4-diols [22]. The profile of the polyphenolic compounds of the cocoa beans will, thus, also influence the antioxidant value of the chocolate obtained therefrom.

Total content of polyphenols, flavonoids, and phenolic acids in the studied samples was determined. The results of total polyphenols were calculated and expressed as gallic acid equivalents, total flavonoid content as quercetin equivalents, and total phenolic acids content as caffeic acid equivalents, as shown in Table 1.

The highest polyphenol content was found in blend chocolate with 90% cocoa solids. On the other hand, the lowest polyphenol content was found in blend chocolate with 100% cocoa solids. The differences between single-origin chocolates in the content of total polyphenols were not significantly important. However, the differences between blend chocolates may be explained by geographical and vegetal factors as the nature of soil, cultivation and fermentation conditions, climate, and cultivar [24]. Because polyphenols are stored in the non-fat cocoa solids, chocolates with a higher cocoa content are considered to be better sources of these compounds, which have a higher antioxidant capacity [25]. Although our results are in accordance with previously reported data [26], they cannot be directly compared due to differences in the solvent used and presentation of the results.

A strong positive correlation (R^2^ = 0.9) was found between the total content of polyphenols and flavonoids, as well as phenolic acids. The total content of flavonoids was high in samples of blend chocolates with 80 and 90% of cocoa solids and in samples of single-origin cocoa chocolate. The lowest content of total flavonoids was found in the blend chocolate with 100% cocoa solids. The content of total phenolic acids correlated with the content of flavonoids (R^2^ = 0.9), as well as the highest and the lowest content of phenolic acids. To this date, it is not entirely clear why phenolic acids usually contribute significantly to the phenolic profile of studied samples, especially chocolates [27]. 

### 2.2. Determination of the Antioxidant Activity of Samples

For a better interpretation of the results, antioxidant activity was evaluated by three different methods. There has still not been established one, single method for the determination of the antioxidant capacity of specific food sample and, therefore, it is recommended to combine at least two or more different methods to provide comprehensive results of the total antioxidant capacity of a food samples [28]. Moreover, all methods used were based on different mechanisms, and this is also the reason why the results from these various methods can be biased. The antioxidant activity of tested samples evaluated by DPPH, ABTS, and FRAP method is shown in Table 2.

The antioxidant activity of all samples measured by DPPH assay varied between 6.09 ± 0.01 g Trolox equivalents (TE)/kg in blend chocolate with 80% cocoa solids and 6.19 ± 0.00 g TE/kg in blend chocolate with 100% cocoa solids. Especially when talking about blend chocolates, there are different cacao cultivars as well as processing parameters used, and all these factors affect the antioxidant capacity of the final products [1]. Our results are in consonance with the results of Belšak et al. [26], where DPPH showed lower results than ABTS test because ABTS radical reacts with both hydrophilic and lipophilic antioxidants while DPPH reacts only with lipophilic ones. In addition, DPPH radical does not react with phenolic acids and, therefore, the antioxidant capacity determined by DPPH and ABTS methods are partially different. The main advantage of the ABTS radical is its high reactivity and, thus better ability to react with a wider range of antioxidant compounds [29]. 

The highest antioxidant activity measured by ABTS assay was shown by the sample of blend chocolate with 90% of cocoa solids, and the lowest activity was found in the sample of 100% cocoa solids’ blend chocolate. These results positively correlated (R^2^ = 0.94) with the total polyphenol and phenolic acids’ content. Chocolate is a very complex matrix consisting of various components, different than polyphenols that could influence the final antioxidant activity of the samples. Moreover, different types of phenolic compounds respond differently in these assays [1]. 

The lowest and the highest activity measured by the FRAP method was found in the same samples as ABTS. Antioxidant capacities of single-origin chocolates evaluated by ABTS and FRAP method were in both cases higher than 100% blend chocolate and comparable to other high-percentage blend chocolates. 

### 2.3. Determination of the Mineral Composition

The contents of all tested 18 elements determined in studied chocolate samples are shown in Table 3. 

All elements can be divided into two groups, to macro elements (Ca, K, Mg, Na, P, and S) and trace elements. The trace elements identified in this study, which were considered by the World Health Organization Expert Committee on Trace Elements in Human Nutrition [30], were further divided into three groups according to their nutritional importance in humans into the following three groups: (1) Essential elements (Cr, Cu, Fe, Mo, and Zn), (2) elements that are probably essential (Mn and Ni), and (3) potentially toxic elements, some of which may have some essential functions at low levels (Al, Cd, Pb, and Sr).

The highest amount of all macronutrients, except Na, was found in the sample of blend chocolate with 100% cocoa mass. This result can be explained by the highest content of cocoa solids among all samples. It is clear that potassium was the most present macronutrient in all the analyzed samples, ranging from 4.6038 to 8.24 mg/g. Potassium helps to maintain normal cell functions, works by preventing an increase in blood pressure in response to excessive sodium intake, and reduces markers of bone turnover and recurrence of kidney stones. No adverse effects of excessive consumption from food alone have been documented [31]. Dark chocolate was a good source of magnesium as well, which was also confirmed by other authors [32]. 

From the essential elements, iron was the metal with highest values found in the samples of blend chocolates with 90 and 100% cocoa solids and single-origin chocolate from Madagascar. The highest amount of Fe, 0.109 mg/g, was found in the sample of 90% chocolate. The same amount of Fe was found in 90% dark chocolate by Cinquanta et al. [32], which makes high-percentage dark chocolate an excellent source of this essential metal. Moreover, the different content of iron together with zinc, phosphorus, and other trace elements can reflect the mineral composition of the soil in which the cocoa beans have been growing, indicating the differences observed due to the origin of cocoa [33]. Zn has an important influence on the immune system and the lack of this mineral can cause atrophy of lymphoid organs [32]. Zinc was not detected in all tested samples. Sample of 100% chocolate was, however, a great source of zinc, with amount of 0.0214 mg/g, comparable to the results of other authors [12]. 

The amount of potentially toxic elements ranged between 0.0002 to 0.0006 mg/g for Pb; Cd was found in low concentrations (0.0002 and 0.0003 mg/g) only in two samples (blend 90% and single-origin Madagascar, respectively). Cd can only be tolerated at extremely low concentrations and it may accumulate in the human body, so the European Commission set the highest permitted levels of Cd in specific cocoa and chocolate products. This limit was set to 0.8 mg/kg in chocolates with 50% or more total dry cocoa solids [34]. None of the tested samples exceeded this limit. Al was found in the same samples as Cd. The acceptable daily intake for Al is 1 mg/kg body weight [35], which would be hardly reached from consumption of dark chocolate in daily life. As was not detected in any evaluated sample.

### 2.4. Volatile Profile and Composition

The aroma of cocoa is one of the most influential quality features, as it is key to the acceptability of cocoa beans and cocoa products such as chocolate. To date, more than 600 aromatic compounds have been identified from cocoa beans and chocolate products. These compounds include heterocyclic compounds, acids, aldehydes and ketones, esters, alcohols, hydrocarbons, nitriles and sulfides, pyrazines, ethers, furans, pyrones, phenols, pyrroles, and many others [13]. Table 4 shows the volatile compounds identified in the chocolate samples.

The predominant compound in all tested samples was acetic acid, varying from 24.78% in sample blend80 to 29.35% of all volatile compounds in sample blend100. Acetic acid in cocoa beans lowers the pH to about 4.5–5.5, which allows enzymatic reactions and subsequent fermentation. Drying and roasting significantly decrease the content of volatile acids, which is one of the prerequisites for the development of a pleasant aroma of the final products. However, if the cocoa beans are dried rapidly, volatile acids are trapped inside the beans, which is undesirable because the high acid content can lead to an unpleasant aroma of both cocoa and chocolate. Low content, on the other hand, may be related to loss of proper chocolate taste [36]. Also, Hinneh et al. [37] identified acetic acid as the most dominant and most sensory active acid in chocolate samples.

The pyrazines are considered the most important group of odorants with chocolate notes [38]. In our samples, tetramethylpyrazine was present in the highest concentrations of pyrazines. The sample with Madagascar origin had the highest content, 285.74 mg/kg of tetramethylpyrazine. The presence of pyrazines in dried fermented beans is practically an indicator of good fermentation process and can predict the quality of the beans [38].

Other very important aromatic compounds are aldehydes, including 3-methylbutanal and 2-methylbutanal, derived from leucine and isoleucine via Strecker degradation. Both compounds are aromatic and their aroma is characterized by cocoa and chocolate tones [37]. The highest amount of 3-methylbutanal and 2-methylbutanal, 51.01 and 32.44 mg/kg, respectively, were identified in the sample of Madagascar origin. From the group of the alcohol compounds, the most present alcohols were isomers of 2,3-butanediol, especially in the sample originating in Madagascar, where their amounts were at the level of 290.34 and 311.54 mg/kg. The presence of 2,3-butanediol, which is quite stable throughout the production chain, is desirable for high-quality cocoa products. In general, it is desirable to obtain a large amount of alcohols in order to obtain a final product characterized by confectionery and floral tones [39]. 

Esters are one of the most important volatile compounds because they are associated with a fruity scent; in chocolate, however, after aldehydes and pyrazines, they play a secondary role in the co-creation of the characteristic chocolate aroma. These compounds are also present in unfermented and roasted beans, but can also be the result of a fermentation process [36]. The most predominant ester, found in all the samples, with highest concentration again in the sample from Madagascar, was phenyl acetate. This compound has been described as commonly found in dark chocolate and its aroma is similar to roses [40].

## 3. Materials and Methods

### 3.1. Experimental Material

The experimental material consisted of three dark chocolates from blend cocoa mass (originating from Mexico, Grenada, Bolivia, and Jamaica) with different percentages of cocoa solids, varying from 80 to 100%, and three single-origin dark chocolates from different regions, namely Madagascar, Vietnam, and Honduras, with 75% cocoa solids. All samples were obtained from the producer (Jordi’s, Hradec Kralove, Czech Republic) in triplicate. 

### 3.2. Sample Extracts’ Preparation

All samples of chocolates were grated into small pieces using a domestic grater. The 0.25 g grated sample was extracted with 20 mL of 80% ethanol for 2 h in a GFL 3005 laboratory shaker (GFL, Burgwedel, Germany). After centrifugation at 4605× *g* (Rotofix 32A, Hettich, Germany) for 10 min and subsequent filtration, the supernatant was used for measurements. All analyses were performed in triplicate.

### 3.3. Total Phenolic Content

The total phenolic content (TPC) was measured using the Folin-Ciocalteu reagent according to the method of Singleton and Rossi [41]. A 100 µL of sample extract were mixed with 100 µL of the Folin-Ciocalteu reagent, 1 mL of 200 g/L Na_2_CO_3,_ and 8.8 mL of distilled water. The absorbance was measured at 700 nm using the UV/Vis spectrophotometer Jenway 6405 (Jenway Limited, Essex, UK) after 30 min of incubation in the dark. Gallic acid (25–250 mg/L; R^2^ = 0.9978) was used as the standard, and the results were expressed as grams of gallic acid equivalents (GAE) per kilogram of chocolate.

### 3.4. Total Flavonoid Content

The total flavonoid content (TFC) was determined using the modified method of Willet [42]. Briefly, 0.5 mL of sample extract was mixed with 100 µL of 100 g/L ethanoic solution of Al_3_Cl, 100 µL of 1 mol/L sodium acetate, and 4.3 mL of distilled water. The absorbance was measured at 415 nm using the Jenway 6405 spectrophotometer after 30 min. Quercetin (0.01–0.50 mg/L; R^2^ = 0.9977) was used as the standard, and the results were expressed as grams of quercetin equivalents (QE) per kilogram of chocolate.

### 3.5. Total Phenolic Acid Content

The total phenolic acid content (TPAC) was determined using the method of Farmakopea Polska [43]. The sample extract (0.5 mL) was mixed with 0.5 mL of 0.5 mol/L HCl, 0.5 mL Arnow′s reagent (100 g/L NaNO_2_ + 100 g/L Na_2_MoO_4_), 0.5 mL of 1 mol/L NaOH, and 0.5 mL of water. The absorbance at 490 nm was then measured using the Jenway 6405 spectrophotometer. Caffeic acid (1–200 mg/L, R^2^ = 0.9996) was used as a standard, and the results were expressed as grams CAE (caffeic acid equivalents) per kilogram of chocolate. 

### 3.6. Antioxidant Activity

#### 3.6.1. DPPH Method

Radical scavenging activity of samples was measured using the method of Sánchez-Moreno et al. [44] with 2,2-diphenyl-1-picrylhydrazyl (DPPH^•^). The 0.4 mL of sample extract was mixed with 3.6 mL of DPPH^•^ solution (0.025 g DPPH^•^ in 100 mL ethanol). After 10 min of resting in the dark, the absorbance of the sample extract was determined using the Jenway 6405 spectrophotometer at 515 nm. Trolox (6-hydroxy-2,5,7,8-tetramethylchroman-2-carboxylic acid) (10–100 mg/L; R^2^ = 0.9881) was used as the standard, and the results were expressed as grams of TEAC (Trolox equivalent antioxidant capacity) per kilogram of chocolate.

#### 3.6.2. ABTS Method

ABTS^+^ radical cation decolorization assay using 2,2′-azino-bis(3-ethylbenzothiazoline-6-sulphonic acid) was determined by the method of Re et al. [45] with minor modifications. Two milliliters of ABTS^+^ solution were mixed with 0.98 mL of PBS (phosphate-buffered saline) and 0.02 mL of sample extract. Absorbance was measured spectrophotometrically on the Jenway 6405 spectrophotometer, 6 min after the addition of sample extract. Trolox (10–100 mg/L; R^2^ = 0.9991) was used as the standard, and the results were expressed as grams of TEAC (Trolox equivalents) per kilogram of chocolate.

#### 3.6.3. Ferric-Reducing Power Method 

Reducing power of samples (FRAP) was determined by the method of Oyaizu [46]. One milliliter of sample extract was mixed with 5 mL PBS (phosphate buffer with pH 6.6) and 5 mL of 10 g/L potassium ferricyanide. The mixture was stirred thoroughly and heated in a water bath at 50 °C for 20 min. After cooling to room temperature, 5 mL of 100 g/L trichloroacetic acid was added. Then 5 mL of the mixture were pipetted into the test tube and mixed with 5 mL of distilled water and 1 mL of 1 g/L ferric chloride solution. The absorbance was measured using the Jenway 6405 spectrophotometer at 700 nm. Trolox (10–100 mg/L; R^2^ = 0.9974) was used as the standard, and the results were expressed as grams of TEAC (Trolox equivalents) per kilogram of chocolate.

### 3.7. Mineral Composition

The content of mineral elements in the samples was determined by inductively coupled plasma optical emission spectrometry (ICP-OES) instrument (Schaumburg, IL, USA). Prior to the determination of the elements, the mineralization of the samples was carried out under wet conditions and elevated pressure. Samples of chocolate, in the amount of 0.1 g, were weighed into Teflon containers and 65% nitric acid in an amount of 8 mL was added to it. The samples thus prepared were sealed in the vessel. Mineralization of the samples was then performed using a Milestone Ethos Ultrawave-One microwave mineralizer (Milestone SRL, Sorisole, Italy). For each group of nine samples, during the microwave mineralization process, the rotor of the system was supplemented with a blank containing only 8 mL of nitric acid alone. The samples were mineralized for approximately one hour using a temperature rise algorithm as specified for biological samples, without exceeding 200 °C. After cooling, the samples were quantitatively transferred to 50-mL flasks and filled up to the mark with redistilled water.

Concentrations of 18 elements (Al, As, Ca, Cd, Cr, Cu, Fe, K, Mg, Mn, Mo, Na, Ni, Pb, Pb, S, Sr, Zn) were determined by optical emission spectrometry with inductively induced plasma using a Thermo iCAP 6500 Spectrophotometer (Thermo Fisher Scientific Inc., Waltham, MA, USA). The detection threshold obtained for each element was not less than 0.01 mg/kg (with an assumed detection capacity of the measuring instrument at a level higher than 1 ppb). The measurement result for each element was compensated to consider the measurement of the elements in the blank. In each case, a 3-point calibration curve was used for each element, with optical correction using the internal standard method in the form of yttrium 89Y and ytterbium 173Yb at concentrations of 2 mg/L and 5 mg/L, respectively. The results were expressed in mg/g.

### 3.8. Volatile Profile and Composition Analysis

Isolation of aromatic compounds was carried out using a headspace solid-phase microextraction (HS-SPME) procedure. A manual SPME holder (Supelco, Bellefonte, PA, USA) with divinylbenzene/carboxene/polydimethylsiloxane (DVB/CAR/PDMS, 50/30 µm) fiber (Supelco) was used to extract the volatile substances. Prior to use, the fiber was conditioned in a gas chromatograph injection port at 250 °C for 1 h. Preparation and extraction of the sample were performed according to Afoakwa et al. [47] with minor modifications. For each extraction, 1 g of homogenized chocolate sample was placed in a 40-mL glass vial with a plastic screw cap and then 15 mL of distilled water were added. Sodium chloride (0.5 g) and 15 µL β-ionone were subsequently added to the sample. β-Ionone was used as an internal standard after checking that it was not primarily present in chocolate, separated well from other volatile substances, was stable at high temperatures, and did not react with water. The prepared samples were subsequently heated in a water bath at 50 °C with temperature control and stirred for 70 min, the first 15 min of which were the equilibration of the components in the vial space and during the remaining time the fiber was introduced and the adsorption of the volatile components to the fiber took place. The SPME fiber was then removed from the vial space and inserted into the gas chromatography–mass spectrometry (GC-MS) injector (230 °C), where it was exposed for 2 min. The separation and identification of volatile compounds were performed on a Shimadzu GC-17A gas chromatograph (Shimadzu, Kyoto, Japan) with a Shimadzu GC-MS QP-5050A mass spectrometer detector. The GC-MS system was equipped with a SLB-5ms GC capillary column (Supelco; 30 m × 0.25 mm internal diameter, 0.25 µm film thickness). Helium was used as a carrier gas at a flow rate of 0.7 mL/min, splitless mode was applied, and the following temperature program performed: (1) Initial temperature 40 °C, (2) rate of 2 °C/min from 40 °C to 180 °C, (3) a rate of 25 °C/min from 180 °C to 300 °C and hold for 1 min. The injector temperature was maintained at 230 °C and the detector temperature at 300 °C. The compounds were simultaneously identified using three different analytical methods: Retention indices (RI) with reference to *n*-alkanes (C8–C24), GC-MS retention times of authentic chemicals, and mass spectra (authentic chemicals and spectral library collection NIST05 (National Institute of Standards and Technology, Gaithersburg, Maryland, USA). Identification was taken as only tentative if it was based only on mass spectral data. Quantification of the volatile compounds was performed on a gas chromatograph Shimadzu 2010 with a flame ionization detector (FID). The column and chromatographic conditions were the same as those previously reported for the GC-MS analysis. The injector temperature was 250 °C and N_2_ was used as a carrier gas (1 mL/min). Data handling was carried out by means of GCsolution 2.3 (Shimadzu Corporation, Kyoto, Japan).

### 3.9. Statistical Analysis

All measurements were performed in triplicate, if not stated differently. Experimental data were evaluated by basic statistical indicators of variability using the Microsoft™ Excel^®^ software (Microsoft Corp., Redmond, WA, USA). The dependency rate between the tested traits was expressed by the linear correlation analysis. Data were also subjected to the Fisher test to compare means. Differences were considered statistically significant at *p* < 0.05.

## 4. Conclusions

Based on the research conducted and results obtained, there were significant differences observed in the total content of polyphenols, flavonoids, and phenolic acids between blend cocoa chocolates with different amounts of cocoa mass. These results suggest that final biological capacity is highly dependent on the characteristics of the raw materials used as their origin, which was not defined for these products. Moreover, our results indicated that 100% chocolate is not the best alternative and the most abundant source of polyphenols and other bioactive substances compared to chocolates with a lower cocoa content. On the other hand, single-origin chocolates, originating from well-defined areas and plantations and marketed as superior and almost luxurious products, did not show significant differences in antioxidant content, and all samples had a high content of these bioactive components. Results for all samples followed their results from the content of bioactive components studied. Moreover, some of the macro- and micronutrients (Mg, K, Fe, and Zn) found in chocolate samples can significantly contribute to healthy nutrition. However, chocolates with specified origin showed differences in aroma compounds compared to the blend chocolates. 

## Figures and Tables

**Table 1:** The content of total polyphenols, flavonoids, and phenolic acids.

Sample	TPC [g/kg]	TFC [g/kg]	TPAC [g/kg]
blend80	14.12 ± 0.62 ^b^	0.31 ± 0.08 ^a^	13.06 ± 0.27 ^b^
blend90	23.58 ± 1.38 ^a^	0.44 ± 0.02 ^a^	16.64 ± 0.01 ^a^
blend100	4.83 ± 0.04 ^c^	0.19 ± 0.15 ^b^	8.46 ± 0.01 ^d^
Origin Madagascar	21.76 ± 0.44 ^a^	0.31 ± 0.04 ^a^	13.48 ± 0.28 ^b^
Origin Vietnam	21.00 ± 0.84 ^a^	0.27 ± 0.00 ^a^	13.84 ± 0.08 ^b^
Origin Honduras	18.78 ± 1.11 ^a,b^	0.37 ± 0.02 ^a^	12.39 ± 0.31 ^c^

Values represent mean ± standard deviation (*n* = 3). Values followed by the same letter in superscript within the same column were not significantly different (*p* < 0.05), according to Fisher’s test. TPC—total phenolic content expressed as gallic acid equivalent, TFC—total flavonoid content expressed as quercetin equivalent, TPAC—total phenolic acids’ content expressed as caffeic acid equivalent.

**Table 2:** The antioxidant activity of samples.

Sample	DPPH [g/kg]	ABTS [g/kg]	FRAP [g/kg]
blend80	6.09 ± 0.01 ^b^	47.19 ± 5.61 ^b,c^	31.20 ± 0.77 ^c^
blend90	6.11 ± 0.01 ^b^	64.43 ± 2.43 ^a^	37.96 ± 1.25 ^a^
blend100	6.19 ± 0.00 ^a^	36.98 ± 0.41 ^d^	19.24 ± 0.84 ^d^
Origin Madagascar	6.17 ± 0.01 ^a^	58.21 ± 0.41 ^b^	31.25 ± 0.26 ^c^
Origin Vietnam	6.11 ± 0.01 ^b^	56.66 ± 2.23 ^b^	35.10 ± 0.28 ^b^
Origin Honduras	6.16 ± 0.00 ^a^	47.93 ± 0.54 ^c^	30.67 ± 0.69 ^c^

Values represent mean ± standard deviation (*n* = 3). Values followed by the same letter in superscript within the same column were not significantly different (*p* < 0.05), according to Fisher’s test. DPPH—2,2-diphenyl-1-picrylhydrazyl, ABTS—2,2′-azinobis(3-ethylbenzothiazoline-6-sulphonic acid), FRAP—ferric reducing power. Results are expressed as g Trolox equivalents (TE) per kg.

**Table 3:** The mineral composition of samples.

Element [mg/g]	Blend80	Blend90	Blend100	Origin Vietnam	Origin Madagascar	Origin Honduras
Al	ND	0.0119	ND	ND	0.0032	ND
As	ND	ND	ND	ND	ND	ND
Ca	0.6110	0.7025	0.9110	0.6280	0.5565	0.4523
Cd	ND	0.0003	ND	ND	0.0002	ND
Cr	ND	ND	ND	ND	0.0003	ND
Cu	0.0021	0.0113	0.0084	0.0041	0.0067	0.0010
Fe	ND	0.1090	0.0444	ND	0.0796	ND
K	5.4550	6.5098	8.2400	5.4900	4.6038	4.7450
Mg	2.1331	2.5327	3.3941	2.1576	1.8112	1.8446
Mn	0.0210	0.0215	0.0206	0.0539	0.0156	0.0168
Mo	ND	ND	0.0005	ND	ND	ND
Na	ND	ND	0.0013	0.0205	ND	ND
Ni	ND	0.0014	ND	0.0023	0.0030	ND
P	2.4418	3.1926	3.8668	2.5468	2.3651	2.4208
Pb	ND	0.0006	ND	0.0002	0.0003	0.0005
S	0.8282	1.0812	1.2092	0.8087	0.7442	0.7987
Sr	0.0081	ND	0.0124	0.0064	ND	0.0033
Zn	0.0074	ND	0.0214	0.0078	ND	0.0039

ND—not detected.

**Table 4:** The aroma composition of samples.

Compound [mg/kg]	Blend80	Blend90	Blend100	Madagascar	Vietnam	Honduras
**Aldehydes**						
Isobutyraldehyde	0.57	1.96	3.15	2.95	1.37	0.43
3-Methylbutanal	11.04	25.84	36.19	51.01	15.36	10.64
2-Methylbutanal	6.50	13.55	18.87	32.44	12.29	8.07
Furfural	ND	8.54	6.43	6.40	ND	2.94
Methional	ND	ND	ND	8.53	ND	1.19
Benzaldehyde	36.26	102.79	135.60	118.84	43.49	45.18
Hyacinthin	31.79	56.45	118.66	118.09	52.15	40.84
Nonanal	12.69	23.85	ND	28.56	13.14	13.96
Caprinaldehyde	ND	ND	1.39	ND	ND	ND
2-Phenyl-crotonaldehyde	0.58	ND	5.13	1.08	3.11	2.97
**Alcanes**						
*n*-Hexane	14.75	73.26	44.95	194.00	17.48	31.50
2,6-Dimethylnonane	ND	ND	ND	ND	ND	1.18
Undecane	ND	ND	ND	11.16	ND	ND
Dodecane	0.82	2.24	ND	ND	ND	ND
Alcohols						
Ethanol	0.31	1.38	1.02	1.79	1.18	0.32
2-Pentanol	2.23	6.48	5.62	11.92	12.70	ND
2,3-Butanediol	26.15	83.94	116.90	290.34	54.86	44.47
2,3-Butanediol	20.37	69.77	71.25	311.54	29.87	24.32
Furfurylalcohol	ND	6.11	15.34	ND	ND	4.66
2-Heptanol	4.19	5.52	9.14	4.98	4.17	2.88
Phenylmethanol	5.31	9.90	11.45	20.73	7.29	4.18
2-Nonanol	ND	ND	30.98	ND	ND	ND
2-Phenylethanol	22.25	53.43	77.41	62.30	44.62	56.92
2-Buthyl-1-oktanol	2.00	11.67	9.32	ND	ND	ND
**Heterocycles**						
Methylpyrazine	ND	ND	ND	ND	ND	0.80
2,5-Dimethylpyrazine	1.99	2.50	10.18	3.62	4.42	3.61
2,3-Dimethylpyrazine	2.75	8.17	8.46	15.11	2.74	3.39
2-Ethyl-6-methylpyrazine	0.47	ND	2.65	ND	ND	15.23
2,3,5-Trimethylpyrazine	15.01	31.40	43.35	81.39	ND	ND
2,6-Dimethyl-3-ethylpyrazine	0.98	6.77	6.36	4.32	2.18	3.10
Tetramethylpyrazine	60.31	93.01	167.66	285.74	103.76	68.40
2,3,5-Trimethyl-6-ethylpyrazine	0.80	1.34	5.07	4.00	2.44	ND
Toluene	0.73	4.75	ND	7.18	ND	1.58
*m*-Cymene	4.10	12.79	10.95	16.44	2.64	3.23
**Esters**						
Methyl vinyl ether	0.59	1.86	2.19	3.11	0.72	0.70
Ethyl acetate	ND	ND	ND	ND	19.12	ND
Isoamyl acetate	ND	ND	ND	ND	ND	3.49
2-Heptyl acetate	0.46	ND	ND	ND	ND	1.36
Benzyl acetate	2.11	6.30	2.59	ND	1.23	ND
Ethyl octanoate	1.65	5.30	4.36	ND	4.71	2.39
Octyl acetate	ND	ND	ND	12.74	ND	ND
1,2,3-Propanetriyl tris(2-ethylbutanoate)	1.78	5.59	5.52	ND	2.79	ND
Phenyl acetate	5.84	13.50	21.10	29.86	13.21	23.39
Isoamyl benzoate	1.83	ND	ND	ND	2.41	ND
**Carboxylic acids**						
Acetic acid	147.51	436.32	585.07	948.75	251.74	204.17
Propionic acid	1.64	ND	ND	ND	ND	ND
**Ketones**						
Acetoine	4.05	6.61	6.79	11.41	4.65	4.22
2-Heptanone	ND	6.88	7.97	ND	ND	2.17
2-Acetylfuran	ND	ND	2.35	ND	ND	0.76
2-Acetylpyrrole	5.47	13.54	23.65	20.89	2.54	5.00
2-Nonanone	3.83	ND	10.17	2.48	2.26	3.42
**Monoterpenes**						
β-Pinene	1.24	5.88	3.80	9.53	1.45	2.18
Myrcene	2.68	8.06	7.09	ND	ND	3.39
Limonene	66.12	225.11	184.95	266.34	46.85	70.20
γ-Terpinene	5.92	15.46	15.17	24.56	ND	4.58
Linalool	5.66	9.36	18.44	ND	6.99	5.08
**Others**						
Dimethyl disulfide	ND	ND	6.23	ND	ND	ND
Dichloromethane	1.02	5.07	5.96	12.88	2.46	2.66
Oxirane, 2-(1,1-dimethylethyl)-3-methyl	1.46	4.70	2.98	2.81	4.16	1.47
Cycloheptatriene	ND	ND	3.90	ND	ND	ND
Isobutylhydrazine	47.14	143.73	94.58	605.95	79.46	46.64

ND—not detected.
